# Address of Dr. Geo. L. Field

**Published:** 1867-07

**Authors:** 


### ADDRESS

### OF DR. GEO. L. FIELD, UPON RETIRING FROM THE PRESIDENCY OF THE MICHIGAN STATE DENTAL ASSOCIATION, AT ADRIAN, JANUARY 22D, 1867.

*Gentlemen of the Michigan State Dental Association:*—In accordance with a custom of this honorable body, it now becomes my duty, as the retiring presiding officer, to deliver before you my valedictory, or farewell address. First thanking you for the honor you have done me, by according to me so honorable a position, and for the lenient manner in which you have looked upon my short-comings in this, to me, novel position, and for the hearty support which you have given me in my endeavors to fulfill those duties devolving upon the office, I will earnestly, and as briefly as possible, say a few words.

Another year has now been numbered 'mid the past, since last we met together in friendly concert to discuss such matters as might come before us, having for their end and aim the good of ourselves and the good of others, in a Dental specialty.

By the good Providence of the Lord, we are again permitted to assemble for the same purpose, and I most sincerely hope that each and every one of you have brought with you some of the fruits of the seed sown one year ago, that we may together judge of its entire worth to *ourselves*, which is equivalent to the good of mankind at large. Let none be afraid to speak, and speak boldly. Some one may have, as he supposes, some *little* new idea, so small indeed that he may not think it worth the mentioning, and yet *that very little thing* may be the key to open the doors of some firmly closed vault in which is hid a mine of wealth. All men are differently endowed. No two minds are exactly alike. All minds are capable of receiving truth, some to a greater and others to a less extent. A man is not the *originator* of *any* new idea. To some this may seem a bold assertion, it is, nevertheless, a *true* one. You may think it hard to be told that, after the labor of days, months, or even of years, upon some piece of mechanism, which shall have, upon its successful completion, a great and good end, confering a great blessing upon mankind, you were not the *real* and *alone* originator. And yet I say, that it is *true*, you are *not*. All improvements, all new ideas, all truth, all *good*, have but one fountain head, and that is *God*. Our minds are but the recipients, or mediums, through which they flow down from *Him*, to this world for the benefit of *all* men. Some men's capacities for receiving these things are greater than others. To some have been given one, to some five, and to some ten talents. It is the *use* that we put these gifts to, that shall qualify the mind; and woe to him that *buries* his in a napkin. To him that little is given, little will be required. This, gentlemen, I mean to apply to him who, having received some new truth or light, shall keep it to *himself*. It is not his own; and if he does not give it to the world, he appropriates to *himself* that which only, in *part*, belongs to him. From how small a thing was deduced the great fact that the world was round—the simple fall of an apple. The seed there sown fell in such soil as was especially adapted to it. Ten thousand men equally as smart as Newton might have stood under an avalanche of them, and nothing more would have occurred to them but that "self-preservation is the first law of nature," and hurried from the spot to save themselves from a premature interment. Speak out then, I say. Let others be the judge as to whether that little offering you lay upon our common altar is of use. You, then, have done *your* duty. We may ponder and investigate as long as we please, and yet we can go but *so* far, and no further. We all know that the blood circulates through the arteries and veins of the body; we know that that circulation is produced by the expansion and contraction of certain muscles of the heart; but who can tell *why* those muscles perform such functions? We know that when they cease to do so, death follows. You know that when an acorn is dropped into the ground a great oak of the forest will come forth from the little shell. Can you tell us why? or two acorns that, to us, may look exactly alike, will bring forth different species of oak, but never, by any possibility, bringing forth any variety but that to which it belongs? The life of a tree comes from the only source from which life *can* come. Little do we know of what influences surround us at all times. In this very room where we are now congregated there is first the atmosphere that we breathe, composed of oxygen, carbon, hydrogen, azote and many other gases; there is light with its variety of colors; there is gravitation connecting us with every orb in the solar system; one cord binding us to the sun, another to the moon, etc. There are electricity, magnetism, galvanism, and many other agents, doubtless, of which we, as yet, know nothing. All are here around us, and yet we never should have known it, but for the deep researches of scientific men—men who, when they took hold of any one given subject, took hold with a *will*, sifted to the bottom. Deep study and application has given us the power of conversing with a friend thousands of miles distant—the agent, electricity. Could Morse, or Franklin tell us what that subtile fluid is that they have brought so perfectly under their control? Science has revealed the fact that our Nation's flag, the Stars and Stripes, (God bless it), is *really* an emblem of *liberty*, for its colors are those contained in that first of all God's gifts to man, i. e., the atmosphere we breathe; the composition of which is, air, aura and ether. Aura is red, air is white, and ether is blue. Who will wonder then that the very breezes should seem to love to kiss its graceful folds as it waves triumphantly over this land of freedom and liberty.

In Dentistry we are but in our infancy. We have a broad field before us. It has been plowed, and we have reaped rich harvests for our labor. But it must be plowed much deeper. We began by replacing, by artificial substitutes, those organs of mastication disease had destroyed. Then a step higher was taken,—we bent our energies to the task of preserving those pearls from *total* destruction, when disease had but partially destroyed them ; but our ultimate end and aim must be to preserve them *from* all disease. It may be, and probably will be, a long time ere we reach such a desideratum. You and I may not see the day, but it will come, and for the good of mankind we can but say "God speed the day."

One other matter I wish to call your attention to, and strongly urge upon your earnest consideration, and that is, the inviting manner in which the *latch string* to this Society is left hanging out. All that is now necessary for any one to gain admittance to assemblies, is to hang a shingle out some where, then step forward, pull the string, pay for his ticket, come in and fall into the arms of those on the inside waiting for him. Now this is all wrong. I opposed it last year, and shall oppose it this, and always. We must all be able to see what must inevitably be the result of such laxity. This is no school of probation ; neither is it one where the rudiments of our profession are taught. There must be a line drawn, and the higher the better, and those who come knocking for admittance must be able to come up to it before being admitted to membership. We took a step forward last year in regard to the reception of students into our different offices in the future. Now let's take a step forward in this matter. It is not the profession or calling that makes the man, but the reverse. And yet I hope to see the day when the simple fact that a man is a *Dentist*, shall confer a certain amount of *dignity* upon him. This can only be done when the walls shall be built so high about us that none can scale them, but must of a necessity pass through the proper door, where strict sentinels shall stand guard, exactingly demanding the magic pass-word, which alone shall admit them as members of the Dental profession; and that pass-word shall be written upon parchment, signed by the Faculty of some *Dental College.*

The fact that so many incompetent and unprincipled men are now allowed to humbug, without limit, the public at large, by pretending to that of which they know nothing, has been the means, to a certain extent, of dimming the lustre of those who are an honor to themselves and to their profession. A movement should and must be made to bring about a reform. Our brethren of Ohio have began the work by an appeal to their State Legislature for protection from the further flooding of their fair State by these charletans and quacks. Let us then awake from our lethargy, buckle on our armor, and in solid column march to the front; then by direct assault, and by the flank, deal such blows as shall soon clear the way for the onward march of light and truth. Keep up that knocking at the door of our State University already commenced, and make an appeal to *our* Legislature for *their* assistance in the matter.

There are many other things on which I should like to speak, but I feel that I have already drawn too deeply upon your time and patience; they will all, probably, be taken up, one by one, discussed and disposed of ere the close of our session. Offering you my apologies for the short-comings of this, my *first* valedictory, for I feel that it is sadly deficient in that which it should contain, trusting that you will still extend that same charity towards me that you have from the time I took this seat, I will, as your President, say farewell.

